# AB0 blood groups and rhesus factor expression as prognostic parameters in patients with epithelial ovarian cancer – a retrospective multi-centre study

**DOI:** 10.1186/s12885-018-4289-6

**Published:** 2018-04-19

**Authors:** Veronika Seebacher, Stephan Polterauer, Alexander Reinthaller, Heinz Koelbl, Regina Achleitner, Astrid Berger, Nicole Concin

**Affiliations:** 1 Department of Gynecology and Gynecologic Oncology, Comprehensive Cancer Center Vienna, Gynecologic Cancer Unit, Medical University of Vienna, Spitalgasse 23, 1090 Vienna, Austria; 2 Department of Gynaecology and Obstetrics, Medical University of Innsbruck, Innrain 52, Christoph-Probst-Platz, 6010 Innsbruck, Austria; 3 Karl Landsteiner Institute for General Gynecology and Experimental Gynecologic Oncology, Franziskanergasse 4a, 3100 St. Pölten, Austria

**Keywords:** Ovarian cancer, AB0 blood groups, Rhesus factor, Prognosis, Survival, Outcome

## Abstract

**Background:**

AB0 blood groups and Rhesus factor expression have been associated with carcinogenesis, response to treatment and tumor progression in several malignancies. The aim of the present study was to test the hypothesis that AB0 blood groups and Rhesus factor expression are associated with clinical outcome in patients with epithelial ovarian cancer (EOC).

**Methods:**

AB0 blood groups and Rhesus factor expression were evaluated in a retrospective multicenter study including 518 patients with EOC. Their association with patients’ survival was assessed using univariate and multivariable analyses.

**Results:**

Neither AB0 blood groups nor Rhesus factor expression were associated with clinico-pathological parameters, recurrence-free, cancer-specific, or overall survival. In a subgroup of patients with high-grade serous adenocarcinoma, however, blood groups B and AB were associated with a better 5-year cancer-specific survival rate compared to blood groups A and 0 (60.3 ± 8.6% vs. 43.8 ± 3.6%, *p* = 0.04). Yet, this was not significant in multivariable analysis.

**Conclusions:**

AB0 blood groups and Rhesus factor expression are both neither associated with features of biologically aggressive disease nor clinical outcome in patients with EOC. Further investigation of the role of the blood group B antigen on cancer-specific survival in the subgroup of high-grade serous should be considered.

## Background

Epithelial cancer of the ovary, the fallopian tube and the peritoneum (EOC) represent the fifth most common cancer type in women [1]. Due to the absence of early symptoms and an effective screening option, the majority of EOC is diagnosed at an advanced stage and hence, patients’ prognosis is poor with only 46% surviving five years [2]. According to the World Health Organization’s cancer statistics, EOC caused the death of 42.749 European women in 2012 [3]. The strongest predictors of survival are tumor stage, volume of residual tumor after surgery, age, histological subtype, amount of ascites, preoperative albumin level, performance status, and family history suggestive of breast/ovarian cancer [4]. Despite all these features, available biomarkers predicting clinical outcomes remain limited [4]. Therefore, there is an unmet need to identify features that reflect the biological and clinical aggressiveness of the tumor.

AB0 blood group antigens have been reported to be expressed in benign and malignant ovarian tumors [5, 6]. The presence of AB0 antigens on epithelial cells of early stage cancer has been suggested to play a role in carcinogenesis by increasing the cell’s resistance to apoptosis [7]. In addition, AB0 blood groups have been associated with tumor progression in several malignancies [8–11]. A recent meta-analysis of 89 studies in 30 cancer sites suggested that blood group A is associated with a higher risk for developing various malignancies such as ovarian cancer [12, 13].

AB0 blood groups and Rhesus factor expression are both routinely assessed for patients with EOC prior to therapy. To date, only few data are available on the association of AB0 blood groups and prognosis in patients with EOC. A small retrospective study on 92 EOC patients suggested that the presence of blood group A is associated with worse prognosis [14]. A second study published recently reported similar results in a retrospective cohort of 256 EOC patients [15]. This cohort comprises only Chinese patients with advanced EOC who received optimal upfront cytoreductive surgery.

To the best of our knowledge, no data is available on the prognostic effect of Rhesus factor expression on EOC patients’ survival. We hypothesized that both the AB0 blood groups and the Rhesus factor expression might be associated with clinical outcomes of patients treated for EOC. To test this hypothesis, we performed a retrospective, multi-center study assessing the impact of AB0 blood groups and Rhesus factor expression on clinico-pathological features and prognosis of patients with EOC.

## Methods

### Patients

Two consecutive series of patients who received primary treatment for EOC between 1995 and 2011 at the Medical University of Vienna (*n* = 456) and the Medical University of Innsbruck (*n* = 375) were included in the present study.

Clinical, pathological and laboratory data were retrospectively extracted from electronic databases. After exclusion of patients with missing clinico-pathological essential data elements, follow-up data, or AB0 blood groups and/or Rhesus factor expression, a total of 518 patients remained eligible for analyses.

### Clinical management

All patients were treated according to international standard of care at the time. Patients in general were scheduled to receive upfront cytoreductive surgery according to FIGO (International Federation of Gynecologists and Obstetricians) guidelines and adjuvant platinum-based combination chemotherapy [16]. If complete upfront resection was not feasible, patients were treated with neoadjuvant chemotherapy followed by interval debulking surgery and adjuvant platinum-based combination chemotherapy. All patients, except those with tumor stages FIGO Ia and tumor grading G1, received a platinum-based combination chemotherapy. All patients were included in a standardized follow-up program for up to ten years at the respective institution. If patients did not present for scheduled follow-up visits administrative personnel or nurses contacted them.

### AB0 blood group antigen and rhesus factor expression determination

As part of clinical routine, serological AB0 blood groups and Rhesus factor expression were determined in patients prior to treatment according to current standard [17]. For blood group typing and antibody screening, an agglutination method was used according to the respective laboratory. The results were uniformly recorded in the patients’ charts.

### Statistics

Values are given as mean (standard deviation (SD)) when normally distributed or as median (interquartile range (IQR)) when distribution was skewed. The distribution of AB0 blood groups and Rhesus factor expression and associations with clinico-pathological parameters were assessed using the Fisher exact and Chi-square tests. Survival probabilities were estimated using the Kaplan-Meier method, and differences between groups were assessed using the Log-rank statistics. The results were analyzed for the endpoints of recurrence-free (RFS), cancer-specific (CSS) and overall survival (OS). RFS times for patients with recurrent disease were censored with the date of diagnosis of the first disease recurrence. Events for CSS were defined as EOC-related death or the date of their last follow-up. Events for OS were defined as EOC-related death, death due to other cause or the date of last follow-up. Univariate survival analyses were performed to identify individual risk factors related to survival. Variables that were found to have a significant association with survival on univariate analyses were included in the multivariable Cox regression models. We analyzed differences between patients with blood group antigen A, B, AB, and 0 expression as well as between patients with Rhesus factor expression. After seeing a trend for shorter survival in patients with blood group antigen A and 0 compared to B and AB expression, we then analyzed differences between patients with either blood group antigen A or 0 expression and patients with blood group antigen B or AB expression. Furthermore, we assessed histological subgroups of high-grade serous (including undifferentiated adenocarcinoma), low-grade serous, mucinous, endometrioid or clear cell adenocarcinoma.

Results of univariate and multivariable survival analyses are given as *p*-value (hazard-ratio [HR] and 95% confidence interval [95%CI]). *P*-values < 0.05 were considered statistically significant; all tests were two-sided. We used the statistical software SPSS 23.0 for Mac (SPSS 23.0.0.0, SPSS Inc., Chicago, IL) for statistical analysis.

## Results

### Patients’ characteristics

A total of 518 patients (*n* = 255 at the Medical University of Vienna, *n* = 263 at the Medical University of Innsbruck) with EOC were eligible for final analyses. The median (IQR) patients’ age at diagnosis was 61.7 (51.5–71.0) years. Distributions for blood groups A, B, AB, and 0 were 212 (40.9%), 76 (14.7%), 33 (6.4%), and 197 (38.0%), respectively. Four hundred thirty-one patients (83.2%) expressed the Rhesus D factor. In 41 patients (19.7%), diagnosis and treatment were done before the year 2000. Three hundred seventy eight patients (73%) were treated by primary cytoreductive surgery followed by adjuvant chemotherapy. Neoadjuvant chemotherapy followed by interval debulking surgery and adjuvant chemotherapy was performed in 101 patients (19.5%). Thirty-three patients (6.4%) and six patients (1.1%) received surgery or chemotherapy alone, respectively. Patients’ clinico-pathological characteristics stratified by AB0 blood groups and Rhesus factor expression are given in Table 1. Neither of them were associated with any clinico-pathological characteristics. In addition, we did not find a relationship between the choice of treatment (i.e. neoadjuvant chemotherapy vs. primary debulking surgery) and the patients’ AB0 blood groups (*p = 0.3*) or Rhesus factor expression (*p = 0.1*). Furthermore, no association was found between AB0 blood groups and Rhesus factor expression (*p* = 0.5). Patients’ characteristics were equally distributed within both study centers (data not shown).

**Table 1:** Characteristics stratified by AB0 blood groups and Rhesus factor expression in 518 EOC patients

	All patients (*n* = 518)	AB0 blood group antigen				*p* value*	Rhesus factor		*p* value*
		A0 (*n* = 212, 40.9%)	B0 (*n* = 76, 14.7%)	AB (*n* = 33, 6.3%)	00 (*n* = 197, 38.1%)		Pos (*n* = 431)	Neg (*n* = 87)	
FIGO tumor stage						*0.4* ^*1*^			*0.7* ^*1*^
Ia	29 (5.6)	15 (51.7)	3 (10.3)	1 (3.4)	10 (34.5)		23 (79.3)	6 (20.7)	
Ib	4 (0.8)	2 (50)	0	0	2 (50)		3 (75)	1 (25)	
Ic	61 (11.8)	23 (37.7)	11 (18)	2 (3.3)	25 (41)		52 (86.7)	8 (13.3)	
IIa	10 (1.9)	1 (10)	2 (20)	2 (20)	5 (50)		8 (80)	2 (20)	
IIb	14 (2.7)	5 (35.7)	2 (14.3)	2 (14.3)	5 (35.7)		11 (78.6)	3 (21.4)	
IIc	20 (3.9)	5 (25)	7 (35)	0	8 (40)		18 (90)	2 (10)	
IIIa	17 (3.3)	6 (35.3)	3 (17.6)	0	8 (47.1)		14 (82.4)	3 (17.6)	
IIIb	29 (5.6)	16 (55.2)	0	3 (10.3)	10 (34.5)		27 (93.1)	2 (6.9)	
IIIc	262 (50.6)	107 (40.8)	40 (15.3)	15 (5.7)	100 (38.2)		218 (83.5)	43 (16.5)	
IV	66 (12.7)	30 (45.5)	7 (10.6)	6 (9.1)	23 (34.8)		51 (77.3)	15 (22.7)	
N/A	6 (1.2)								
Histologic Subtype						*0.4* ^*2*^			*0.2* ^*2*^
High-grade serous	310 (59.8)	125 (40.3)	39 (12.6)	19 (6.1)	127 (41)		252 (81.6)	57 (18.4)	
Low-grade serous	50 (9.7)	25 (50)	10 (20)	1 (2)	14 (28)		45 (90)	5 (10)	
Mucinous	34 (6.6)	10 (29.4)	7 (20.6)	4 (11.8)	13 (38.2)		30 (90.9)	3 (9.1)	
Endometrioid	72 (13.9)	30 (41.7)	9 (12.5)	6 (8.3)	27 (37.5)		59 (81.9)	13 (18.1)	
Clear cell	12 (2.3)	7 (58.3)	2 (16.7)	0	3 (25)		10 (83.3)	2 (16.7)	
Mixed epithelial	40 (7.7)	15 (37.5)	9 (22.5)	3 (7.5)	13 (32.5)		33 (82.5)	7 (17.5)	
Postoperative residual tumor						*0.2* ^*3*^			*0.9* ^*3*^
none	229 (44.2)	83 (36.2)	36 (15.7)	9 (3.9)	101 (44.1)		190 (83.3)	38 (16.7)	
≤ 1 cm	66 (12.7)	26 (39.4)	12 (18.2)	6 (9.1)	22 (33.3)		55 (83.3)	11 (16.7)	
> 1 cm	117 (22.6)	47 (40.2)	16 (13.7)	10 (8.5)	44 (37.6)		93 (80.2)	23 (19.8)	
N/A	106 (20.5)								

*Pearson Chi-Square test; *N/A* not available; ^1^FIGO III and IV vs. FIGO I and II; ^2^high-grade serous and mixed epithelial vs. low-grade serous vs. mucinous vs. endometrial and clear cell adenocarcinoma; ^3^residual vs. no residual tumor

Within a median (IQR) follow up period of 38.7 (19.3–68.8) months, 306 patients (59.1%) suffered from recurrent disease with a median time to recurrence of 15.1 months (95% CI: 13.7–16.4). At the time of last follow-up, 200 patients (38.6%) were free of disease, 41 patients (7.9%) had stable disease and 46 patients (8.9%) had progressive disease. Two hundred eight patients (40.2%) died due to EOC and 23 (4.4%) due to other causes. Median OS was 68.8 months (95% CI: 57.7–79.9)

### Association of AB0 blood groups and rhesus factor expression with survival in all patients

**Fig. 1:**
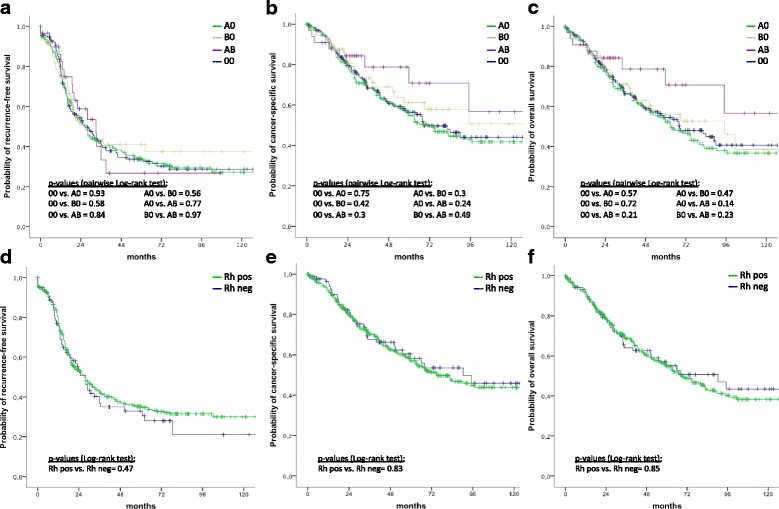
Kaplan Meier survival curves according to AB0 blood group and Rhesus factor expression. Probabilities of recurrence-free survival (**a**, **d**), cancer-specific survival (**b**, **e**), and overall survival (**c**, **f**) stratified by AB0 blood group antigen and Rhesus factor expression in 518 women treated for EOC (A0: *n* = 212; B0: *n* = 76; AB: *n* = 33; 00: *n* = 197)

Univariate survival analyses revealed no association between blood groups 0, A, B, or AB and RFS, CSS or OS (see Fig. 1a-c).

Furthermore, no association could be found between pooled blood groups (i.e. blood groups A versus non-A) and RFS, CSS or OS (*p* = 0.7, *p* = 0.4, *p* = 0.3, respectively). These results did not change when analyzing the subgroup of patients older than 62 years only (*p* = 0.7, *p* = 0.4, *p* = 0.2, respectively).

Similarly, in the subgroup of patients with advanced EOC FIGO tumor stages III and IV, there was no association between blood group A and RFS, CSS or OS when compared to other non-A blood groups (*p* = 0.9, *p* = 0.5, *p* = 0.5, respectively). In addition, we did not find an association between survival and other pooled blood groups (i.e. blood groups B versus non-B; blood groups A and B versus 0, *p*-values not given).

There was no association between the expression of Rhesus factor and RFS, CSS or OS (see Fig. 1d-f).

### Association of AB0 blood groups and rhesus factor expression with survival in patients with high-grade serous adenocarcinoma

**Fig. 2:**
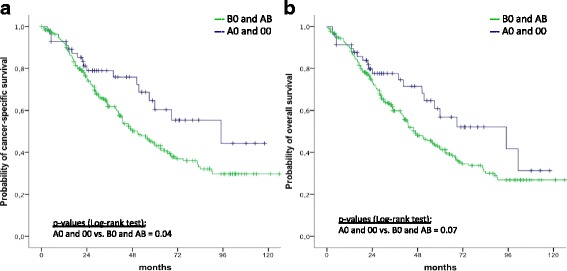
Kaplan Meier survival curves in high grade serous EOC patients. Probabilities of cancer-specific (**a**) and overall survival (**b**) in 310 patients with high grade serous EOC stratified by blood group antigens A0 and 00 (*n* = 252) versus B0 and AB (*n* = 58)

In patients with high-grade serous adenocarcinoma (*n* = 310), Kaplan Meier analysis revealed that patients with blood groups B and AB had a better 5-year CSS than patients with blood groups A and 0 (60.3 ± 8.6% vs. 43.8 ± 3.6%, *p* = 0.04). However, after adjustment for the effects of standard prognostic parameters, in multivariable analysis, only residual tumor, histological grading, FIGO tumor stage, and the median age at diagnosis, but not blood groups, were associated with CSS. Results of uni- and multivariable analyses are given in Table 2. No association was noted between blood groups and OS or RFS within this subgroup (*p* = 0.07 and *p* = 0.1, respectively). Figure 2 provides Kaplan-Meier curves for pooled blood groups (B and AB vs. A and 0) with respect to CSS and OS. Other than that, we did not find any other pooled AB0 subgroups to be associated with survival in patients with high—grade serous adenocarcinoma.

**Table 2:** Uni- and multivariable cancer-specific survival analyses in 310 patients with high-grade serous EOC

	Univariate analyses^a^			Multivariable analyses^a^		
	HR	95% CI	*P*-value	HR	95% CI	*P*-value
Blood groups						
A & 0 vs. B & AB	1.6	1.1–2.6	0.04	1.5	0.8–2.6	0.1
FIGO tumor stage						
IV vs. III vs. II vs. I	1.7	1.3–2.2	< 0.001	1.7	1.2–2.4	0.003
Residual tumor						
> 1 cm vs. > 0 ≤ 1 cm vs. 0 cm	1.9	1.5–2.4	< 0.001	1.8	1.4–2.2	< 0.001
Median age						
> 61.7 vs. ≤ 61.7 years	1.7	1.2–2.4	< 0.001	1.5	1.1–2.2	0.02

^a^Cox Regression model; *HR* hazard ratio; *CI* confidence interval

Rhesus factor expression was not associated with any outcomes in this subgroup (*p* = 0.6, *p* = 0.1, and *p* = 0.2 for RFS, CSS, and OS, respectively).

## Discussion

We evaluated the prognostic value of AB0 blood groups and Rhesus factor expression in a large multicenter study of patients with EOC. We rejected our hypothesis and found no association of either AB0 blood groups or Rhesus factor expression with clinical outcomes. We found that blood group antigen B was associated with a prolonged CSS in patients with serous or undifferentiated adenocarcinoma. However, this effect was not independent of that of standard prognostic parameters in multivariable analysis.

Unlike the findings of two previous studies we could not validate that patients with blood group A have shorter survival than those with other blood groups [14, 15]. As Zhou et al. included patients with advanced EOC who underwent optimal primary debulking surgery only, and as the authors pointed out that the effect was stronger in elder patients, we performed analyses in the subgroups of patients with FIGO III and IV and in patients older than 62 years of age. Even in these subgroups, we could not find an association between blood group A and the patients’ survival outcome. We assume the inconsistency might be related to the differences in the patients’ collectives, as Zhou et al. included Chinese patients only in their study [18]. Furthermore, in the study of Zhou et al., the effect of blood group A was not adjusted for the effect of residual tumor after surgery, one of the strongest known prognostic parameters [19]. Unfortunately, as the study by Marinaccio et al. was published in Italian language only, we could not assess further details [14].

Interestingly, we found associations of AB0 blood groups with survival in the subgroup of patients with high-grade serous carcinoma. We found that patients with blood groups B and AB had a significantly longer CSS compared to those with blood groups A or 0. Yet, this effect was no longer seen after adjustment for the effects of established prognostic parameters. We did not perform subgroup analyses of the effect of AB0 blood groups on survival in patients with low-grade serous, mucinous, endometrioid or clear cell carcinoma as the number of patients within these subgroups was too small.

The genomic structure of AB0 genes consists of seven exons that span approximately 19 kb of genomic DNA on the long arm of chromosome 9, band q34 [20]. Several studies could show that 48 to 59% of epithelial ovarian tumors display genetic imbalance on the long arm of chromosome 9 [21, 22]. These changes were even more common in serous, undifferentiated and late stage tumors. Alterations have been observed most frequently in sub-chromosomal region 9q32-q34 that has, therefore, been suggested to be a potential tumor suppressor locus. Furthermore, differences in blood group antigen expression on EOC tumor cells could be observed between histologic subgroups [6]. Compared to benign and low grade mucinous carcinoma, blood group antigens were less abundant in serous carcinoma tumor cells. The presence of AB-H antigens on uro-epithelial cells in women has been associated with a reduced risk of recurrent urinary tract infections. A protective effect of blood group antigens by preventing binding of microbial pathogens has been postulated [23]. AB-H antigens are furthermore found having a role in cell membrane integrity, cell adhesion, membrane transportation of molecules, and acting as receptors for extracellular ligands and enzymes [24]. It was postulated that the loss of blood group antigens results in improved tumor cell ability to move and circulate through the body because of a loss of cell adhesion proteins [25]. A previous study investigated the expression of AB-H blood group antigens on various sites of EOC, but could not detect a difference between the primary tumor and metastatic site [25]. Moreover, one study investigating 130 EOC tumors could not observe an association between expression of AB-H antigens on the tumor cells and the patients’ survival [26]. The exact role of AB0 blood group antigens and their expression or absence on tumor cells is yet unclear.

We also investigated the role of Rhesus factor expression as prognostic parameter in patients with EOC. The risk of developing various malignancies such as skin, esophageal, breast, lung, and endometrial cancer has been linked to a higher probability of being Rhesus factor-negative [27]. In the present study, we could not show any association between Rhesus factor expression and clinico-pathological features or survival outcomes. Similar results were shown in patients with bladder cancer [11].

To our knowledge, this is the largest study investigating the association between AB0 blood groups and survival in patients with EOC. Furthermore, this is the first study to investigate differences in histological subtypes and the role of Rhesus factor expression. The distribution of blood groups within the present study correlates with blood group distribution in the general Austrian population [28]. Patients’ characteristics were distributed equally in both study centers and pathological and survival outcomes are representative for EOC patients. Although multicenter data acquisition offers the opportunity to narrow study bias due to patient selection, the present study has several limitations inherent to its retrospective nature, such as incomplete data acquisition and heterogeneity in care. Differences in pathologic and surgical procedures may have masked small effects. Finally, information about blood group antigen expression on tumor cells is missing.

## Conclusions

In conclusion, our findings do not suggest any prognostic value for AB0 blood groups or Rhesus factor expression in patients with EOC. We could not validate the findings of previous studies, but assume this is due to differences in patient cohorts and treatment modalities. Our study shows that in patients with high grade serous EOC, the presence of blood group antigen B is associated with a prolonged CSS. Yet, this requires further validation as this was only in univariate analysis and there was no association with other outcomes.

## References

[1] Torre LA, Bray F, Siegel RL, Ferlay J, Lortet-Tieulent J, Jemal A (2015). Global cancer statistics 2012. CA Cancer J Clin.

[2] Howlader N, Noone AM, Krapcho M, Garshell J, Miller D, Altekruse SF, et al (eds). SEER Cancer Statistics Review, 1975–2012, National Cancer Institute. Bethesda, MD. [SEER Web site]. April 1, 2015. Available at: http://seer.cancer.gov/csr/1975_2012/. Accessed 1 June 2016.

[3] Ferlay J, Soerjomataram I, Ervik M, Dikshit R, Eser S, Mahters C, et al. GLOBOCAN 2012 v1.0, Cancer Incidence and Mortality Worldwide: IARC CancerBase No. 11 [Globocan Web site]. 2013. Available at: http://globocan.iarc.fr. Accessed 3 June 2016.

[4] Barlin JN, Yu C, Hill EK, Zivanovic O, Kolev V, Levine DA (2012). Nomogram for predicting 5-year disease-specific mortality after primary surgery for epithelial ovarian cancer. Gynecol Oncol.

[5] Rubin SC, Finstad CL, Hoskins WJ, Provencher D, Federici MG, Lloyd KO (1991). Analysis of antigen expression at multiple tumor sites in epithelial ovarian cancer. Am J Obstet Gynecol.

[6] Vitiazeva V, Kattla JJ, Flowers SA, Linden SK, Premaratne P, Weijdegard B, et al. The 0-linked glycome and blood group antigens AB0 on mucin-type glycoprotein in mucinous and serous epithelial ovarian tumors. PLoS One. June 2015; 10.1371/journal.pone.0130197. 10.1371/journal.pone.0130197 PMC4468167 26075384

[7] Le Pendu J, Marionneau S, Cailleau-Thomas A, Rocher J, Le Moullac-Vaidye B, Clément M (2001). ABH and Lewis histo-blood group antigens in cancer. APMIS.

[8] Kaffenberger SD, Morgan TM, Stratton KL, Bloachie AM, Barocas DA, Chang SS, et al. AB0 blood group is a predictor of survival in patients undergoing surgery for renal cell carcinoma. BJU Int. 2012;110 (11 Pt B):E641–E646. 10.1111/j.1464-410X.2012.11366.x. Epub 2012 Sep 7. 10.1111/j.1464-410X.2012.11366.x 22958439

[9] Rhabari NN, Bork U, Hinz U, Leo A, Kirchberg J, Koch M (2012). AB0 blood group and prognosis in patients with pancreatic cancer. BMC Cancer.

[10] Klatte T, Xylinas E, Riecken M, Kluth LA, Rouprêt M, Pycha A (2014). Impact of AB0 blood type on outcomes in patients with primary nonmuscle invasive bladder cancer. J Urol.

[11] Engel O, Soave A, Peine S, Kluth LA, Schmid M, Shariat SF (2015). The impact of the AB0 and the rhesus blood group system on outcomes in bladder cancer patients treated with radical cystectomy. World J Urol.

[12] Zhang BL, He N, Huang YB, Song FJ, Chen KX (2014). AB0 blood groups and risk of cancer: a systematic review and meta-analysis. Asian Pac J Cancer Prev.

[13] Gates MA, Wolpin BM, Cramer DW, Hankinson SE, Tworoger SS (2011). AB0 blood group and incidence of epithelial ovarian cancer. Int J Cancer.

[14] Marinaccio M, Traversa A, Carioggia E, Valentino L, Coviello M, Salamanna S (1995). Blood groups of the AB0 system and survival rate in gynecologic tumors. Minerva Ginecol.

[15] Zhou J, Yang LC, He ZY, Li FY, Wu SG, Sun JY (2015). Prognostic impact of AB0 blood group on the survival in patients with ovarian cancer. J Cancer.

[16] Benedet JL, Bender H, Jones H, Ngan HY, Pecorelli S (2000). FIGO staging classifications and clinical practice guidelines in the management of gynecologic cancers. FIGO committee on gynecologic oncology. Int J Gynaecol Obstet.

[17] Bethesda MD (2012). Standards for blood banks and transfusion services. American Association of Blood Banks.

[18] Farhud DD, Zarif YM (2013). A brief history of human blood groups. Iranian J Publ Health.

[19] Du Bois A, Reuss A, Pujade-Lauraine E, Harter P, Ray-Coquard I, Pfisterer J (2009). Role of surgical outcome as prognostic factor in advanced epithelial ovarian cancer: a combined exploratory analysis of 3 prospectively randomized phase 3 multicenter trials: by the Arbeitsgemeinschaft Gynaekologische Onkologie Studiengruppe Ovarialkarzinom (AGO-OVAR) and the Groupe d’Investigateurs Nationaux Pour les Etudes des Cancers de l’Ovaire (GINECO). Cancer.

[20] Hakomori S (1999). Antigen structure and genetic basis of histo-blood groups a, B and 0: their changes associated with human cancer. Biochim Biophys Acta.

[21] Cliby W, Ritland S, Hartman L, Dodson M, Halling KC, Keeney C (1993). Human epithelial ovarian cancer allelotype. Cancer Res.

[22] Schultz DC, Vanderveer L, Buetow KH, Boente MP, Ozols RF, Hamilton TC (1995). Characterization of chromosome 9 in human ovarian neoplasia identifies frequent genetic imbalance on 9q and rare alterations involving 9p, including CDKN2. Cancer Res.

[23] Stapleton A, Nudelman E, Clausen H, Hakomori S, Stamm WE (1992). Binding of uropathogenic Escherichia coli R45 to glycolipids extracted from vaginal epithelial cells is dependent on histo-blood group secretor status. J Clin Invest.

[24] Reid ME, Mohandas N (2004). Red blood cell blood group antigens: structure and function. Semin Hematol.

[25] Ichikawa D, Handa K, Hakamori S (1998). Histo- blood group a/B antigen deletion/reduction vs. continuous expression in human tumor cells as correlated with their malignancy. Int J Cancer.

[26] Welshinger M, Finstad CL, Venkatraman E, Federici MG, Rubin SC, Lewis JL Jr, et al. Expression of a, B, and H blood group antigens in epithelial ovarian cancer: relationship to tumor grade and patient survival. Gynecol Oncol 1996;62:106–112. 10.1006/gyno.1996.0198 8690281

[27] Huang CH, Ye M (2010). The Rh protein family: gene evolution, membrane biology, and disease association. Cell Mol Life Sci.

[28] Austrian Red Cross – AB0-Blutgruppen-Häufigkeit [Austrian Red Cross Web site]. Available at: www.roteskreuz.at. Accessed 3 June 2016.

